# Cabergoline-Induced Gambling Causing Increased Adrenal Crisis Episodes

**DOI:** 10.1210/jcemcr/luaf263

**Published:** 2025-11-10

**Authors:** Mercedes Martinez-Gil, Shadee Aghel, Tshibambe N Tshimbombu, Kevin C J Yuen

**Affiliations:** Department of Internal Medicine, Creighton University School of Medicine, Phoenix, AZ 85012, USA; Creighton University School of Medicine, Creighton University, Phoenix, AZ 85012, USA; Department of Neurology, Barrow Neurological Institute, Phoenix, AZ 85013, USA; Barrow Pituitary Center, Barrow Neurological Institute, Phoenix, AZ 85013, USA; Department of Medicine, University of Arizona College of Medicine, Phoenix, AZ 85004, USA; Department of Medicine, Creighton University School of Medicine, Phoenix, AZ 85012, USA

**Keywords:** acromegaly, dopamine agonists, cabergoline impulse control disorders, pathological gambling

## Abstract

Dopamine agonists have been used in the treatment of acromegaly, but they are modestly effective and are rarely associated with impulse control disorders (ICDs). We present the case of a 34-year-old woman with acromegaly who underwent transsphenoidal resection for an invasive pituitary macroadenoma, followed by the development of panhypopituitarism. Postoperative imaging revealed residual tumor, and her insulin-like growth factor I levels remained elevated, indicating disease persistence. She subsequently received salvage radiosurgery and was treated with lanreotide and cabergoline, achieving biochemical control of her disease. The patient later reported frequent adrenal crisis episodes with worsening anxiety. Four years after initiating cabergoline, she disclosed impulse control behaviors, including pathological gambling, which had resulted in financial stress and emotional strain. Two weeks after discontinuing cabergoline, she had complete resolution of her ICD symptoms with improved anxiety levels and fewer adrenal crisis episodes. In this case, cabergoline-induced gambling contributed to psychosocial stress that likely exacerbated her adrenal crisis episodes. Our case emphasizes the need for routine behavioral screening in patients treated with dopamine agonists. Early identification and discontinuation or dose reductions of dopamine agonists is recommended to allow for ICD resolution and consequently, reducing the risk of exacerbating adrenal crisis episodes.

## Introduction

Dopamine agonists (eg, bromocriptine and cabergoline), the mainstay of treatment for prolactinomas [1], have also been used as medical therapy for acromegaly patients [2]. These agents exert their effects by binding to the dopamine D2 receptors, which are expressed in most somatotroph adenomas [3]. However, their efficacy in normalizing insulin-like growth factor I (IGF-1) levels is modest, with approximately one-third of patients achieving normalization when used as monotherapy [2]. These agents are beneficial for patients with mild disease, as adjunctive therapy when surgery fails to induce disease remission, or as combination therapy with somatostatin analogs [4] or pegvisomant [5]. Despite their therapeutic effects, dopamine agonists are associated with behavioral side effects, including impulse control disorders (ICDs). Impulsivity is defined as the tendency toward risky and unplanned behavioral responses to internal and external stimuli, with little understanding of the unpleasant consequences to oneself or others [6]. Furthermore, ICDs are defined as behaviors resulting from an inability to resist temptations, urges, or drives, which can be harmful to society or to the individual. Examples of reported ICDs include pathological gambling, hypersexuality, compulsive shopping, punding, and binge eating [7]. These side effects, while rare, are well-documented in association with dopamine agonist therapy [8, 9] and can cause significant lifestyle changes unbeknownst to the patient, their partners, or their caregivers.

Here, we present a case of a patient with acromegaly treated with cabergoline who, over years of being on treatment, developed ICD symptoms that she was not aware of herself and did not disclose to her family and her provider. In many instances, these side effects can be overlooked or incorrectly attributed to underlying psychiatric or social factors. In our patient, her ICD manifested as pathologic gambling, driven by a compulsive urge to acquire money, which led to significant psychosocial stress and heightened anxiety. This behavior very likely contributed to an increased number of potentially life-threatening adrenal crisis episodes. Early recognition and timely intervention of this phenomenon could help alleviate psychosocial distress and potentially lower the risk of adrenal crisis episodes.

## Case Presentation

**Figure 1.**
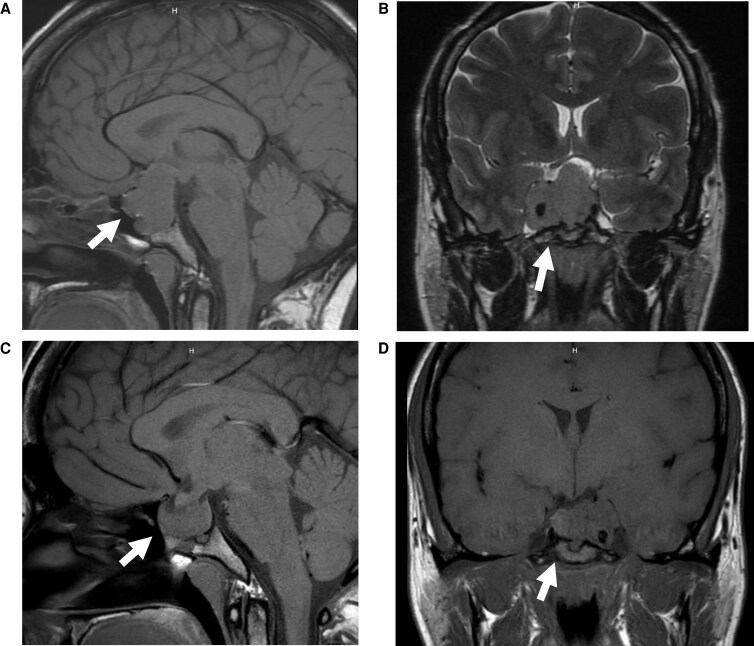
Pre- and postoperative MRI of the pituitary macroadenoma. Panel A, Preoperative sagittal MRI demonstrating a 3.5 × 3.2 × 2.8 cm sellar/suprasellar lesion with mass effect on the optic chiasm, invasion of the right cavernous sinus, and extension through the sellar floor into the sphenoid sinus. Panel B, Preoperative coronal MRI demonstrating tumor invasion into the right cavernous sinus. Panel C, Residual adenoma within the sella and extending posteriorly toward the prepontine cistern. Panel D, Postoperative coronal MRI showing decreased bulk of residual pituitary tumor in the right cavernous sinus.

A 34-year-old woman was diagnosed with acromegaly in 2017. Brain magnetic resonance imaging (MRI) revealed a 3.5 × 3.2 × 2.8 cm (1.38 × 1.26 × 1.10 in) pituitary macroadenoma with suprasellar extension compressing the optic chiasm and bilateral cavernous sinus invasion (Fig. 1). She underwent transsphenoidal surgery (TSS), achieving partial tumor resection and decompression of the optic chiasm, with significant improvement in her vision. Due to the extensive cavernous sinus invasion, only a subtotal resection was feasible in order to preserve normal pituitary function and avoid vascular or cranial nerve injury. Pathology was consistent with an acidophil stem cell adenoma, showing immunoreactivity for both growth hormone and prolactin.

## Diagnostic Assessment

Following surgery, the patient developed panhypopituitarism, including secondary adrenal insufficiency, secondary hypogonadism, and secondary hypothyroidism. Random cortisol obtained postoperatively was 2.7 µg/dL (74.5 nmol/L) (normal: 6.2-19.4 µg/dL [171-535 nmol/L]). Thyroid-stimulating hormone was 1.22 µIU/mL (1.22 mIU/L) (normal: 0.4-4.0 µIU/mL [0.4-4.0 mIU/L]) with a low free thyroxine of 0.69 ng/dL (8.9 pmol/L) (normal: 0.8-1.8 ng/dL [10-23 pmol/L]) and a normal total triiodothyronine of 2.12 ng/mL [3.26 nmol/L] (normal: 0.8-2.0 ng/mL [1.2-3.1 nmol/L]). Estradiol was < 25 pg/mL (<92 pmol/L) (normal follicular phase: 30-120 pg/mL [110-440 pmol/L]) with inappropriately low gonadotropins, follicle-stimulating hormone 2.8 mIU/mL (2.8 IU/L) (normal follicular phase: 3.5-12.5 mIU/mL [3.5-12.5 IU/L]), and luteinizing hormone 0.60 mIU/mL (0.60 IU/L) (normal follicular phase: 2.4-12.6 mIU/mL [2.4-12.6 IU/L]), confirming central hypogonadism. She was treated with hydrocortisone 15 mg each morning and 5 mg each afternoon, along with levothyroxine 75 µg daily.

**Figure 2.**
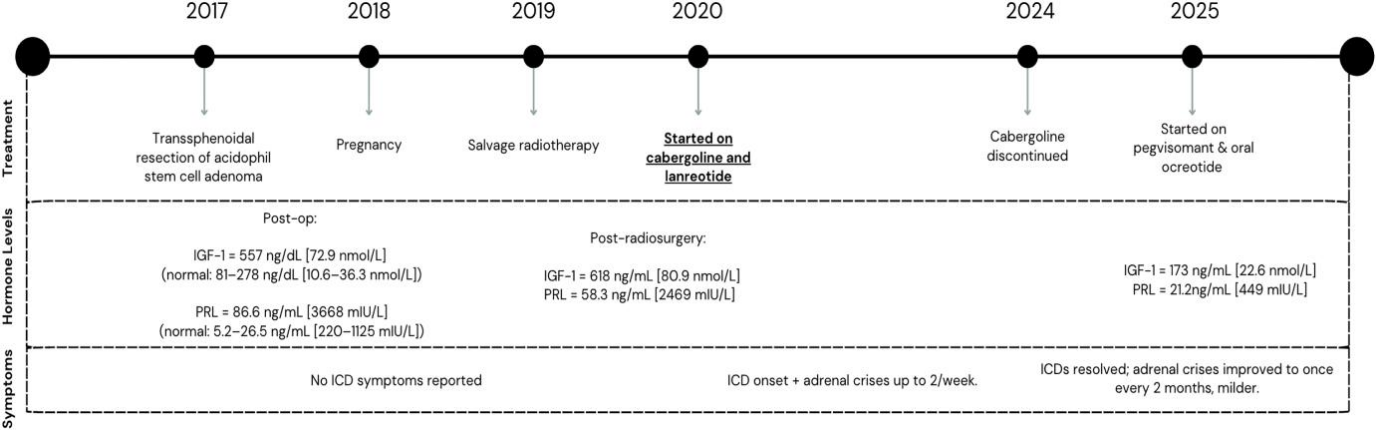
Timeline of clinical course, treatments, hormone levels, and symptoms from diagnosis in 2017 through 2025.

Postoperative prolactin levels remained elevated at 86.6 ng/mL (3668 mIU/L) (normal: 5.2-26.5 ng/mL [220-1125 mIU/L]), and a residual tumor measuring 1.8 × 1.8 cm (0.71 × 0.71 in) was noted at the 3-month postoperative MRI in the right cavernous sinus (Fig. 2). Her IGF-I level at that time was also elevated at 557 ng/dL (72.9 nmol/L) (normal: 81-278 ng/dL [10.6-36.3 nmol/L]), indicating disease persistence. Because of her pathology findings and postoperative MRI findings, radiation therapy was considered but this therapeutic option was ultimately deferred due to the patient's desire for pregnancy in the near future. She successfully became pregnant 4 months after surgery.

## Treatment

After her child was born in 2018, the patient underwent salvage radiosurgery in 2019. Postoperative IGF-I and prolactin levels continued to remain elevated (618 ng/mL [80.9 nmol/L] and 58.3 ng/mL [2469 mIU/L], respectively), prompting the initiation of medical therapy with lanreotide 90 mg every 4 weeks. Because of persistently elevated IGF-I level despite treatment with lanreotide, cabergoline 0.5 mg twice weekly was added in 2020. This combination of medical therapies successfully normalized her IGF-I levels.

From 2020 to 2024, despite stable biochemical control of her disease, the patient experienced an increased frequency of adrenal crisis episodes—up to twice weekly. Typical symptoms included fatigue, dizziness, nausea, lightheadedness, and occasional syncope, which prompted several emergency department visits where she required intravenous hydrocortisone (25 mg every 8 hours). Most episodes, however, were managed independently at home with stress-dose steroids, during which she doubled or tripled her baseline hydrocortisone regimen of 15 mg in the morning and 5 mg in the afternoon. These crises were not initially associated with any identifiable precipitating factors during this period.

In 2024, she eventually disclosed a history of progressive impulse control symptoms. She described her symptoms as beginning with online resale activities for profit, escalating to daily cryptocurrency gambling, which led to financial strain, interpersonal conflict, and worsening anxiety. She noted that her symptoms began shortly after starting cabergoline and that she had initially failed to recognize the severity of her obsession with online gambling and money-making. These changes in behavior overlapped, timewise, with the above-mentioned increase in adrenal crisis episodes.

## Outcome and Follow-Up

The patient was unaware of the link between dopamine agonists and ICDs and at the same time, felt too embarrassed to disclose her behaviors to her provider and family members. It was only after her mother had learned of the association through a friend and conducted further research online that they suspected cabergoline as the contributing factor. Following this realization, the patient discontinued cabergoline and within 2 weeks, her compulsive behaviors resolved completely.

Over the subsequent months, she reported significantly reduced anxiety and a marked decline in adrenal crisis frequency. She experienced only occasional episodes, approximately once every 2 months during intercurrent illness, and the symptoms were notably milder. In addition, her hydrocortisone dose has been maintained on her prior baseline regimen of 15 mg in the morning and 5 mg in the afternoon. The patient has since remained free from gambling and other high-risk behaviors. She is currently treated with pegvisomant 10 mg every other day and oral octreotide 40 mg twice daily, with her most recent IGF-1 level measured at 173 ng/mL (22.6 nmol/L) (reference range: 81-278 ng/mL [10.6-36.3 nmol/L]), indicating ongoing biochemical control of her acromegaly.

## Discussion

Dopamine agonist therapy has been reported in the literature to be associated with a range of behavioral issues, particularly ICDs [10, 11]. The defining feature of ICDs is failing to resist impulses to engage in a pleasurable activity that is harmful to self or others. These behaviors can include pathological gambling, hypersexuality, compulsive shopping, punding, and binge eating [12].

The specific prevalence of ICDs related to cabergoline use in patients with acromegaly is not well established, likely due to under-reporting. However, studies on dopamine agonists as a class suggest that the prevalence of ICDs can range from 10% to 17% in patients with Parkinson disease [13]. However, it is noteworthy to mention that the doses of dopamine agonists used in Parkinson disease are significantly higher than those used in the treatment of acromegaly. Despite the lower doses typically used, there have been several case reports of ICDs developing in patients treated with cabergoline for hyperprolactinemia, which utilizes doses that are comparable to those used in acromegaly [7, 11].

The behavioral side effects of dopamine agonists are primarily attributed to their stimulation of dopamine D2 receptors within the mesolimbic pathway, a neural circuit critical for reward-driven behaviors [10, 11]. Another proposed mechanism involves the suppression of prolactin by dopamine agonists, leading to the restoration of sex steroid secretion. Elevated levels of sex steroid hormones, such as testosterone, are thought to play a significant role in the development of hypersexuality, further contributing to the behavioral changes observed in affected individuals [11].

In patients with existing adrenal insufficiency, the onset of ICDs can precipitate adrenal crises through multiple pathways. Emotional stress and anxiety, which are often heightened in the context of ICDs, are well-recognized triggers for adrenal crisis because it raises cortisol demands beyond baseline replacement needs [14]. In addition, the impulsivity and disorganized behaviors inherent to ICDs may result in missed medication doses, irregular dosing schedules, or neglect of sick-day rules, all of which are established precipitants of adrenal crisis [15]. The combination of heightened stress and impaired adherence may create a synergistic risk for adrenal crisis.

ICDs associated with cabergoline therapy generally improve after discontinuation of the drug, although the time to resolution can vary from days to several weeks, and in rare cases, symptoms may persist longer or require psychiatric intervention [9, 12]. In our patient, her compulsive behaviors and anxiety resolved within 2 weeks of discontinuation, which is consistent with the rapid improvement reported in most case series.

Our case underscores the importance of counseling patients to recognize and report ICDs to their providers and their potential to exacerbate serious underlying conditions such as adrenal insufficiency in those with concurrent adrenal insufficiency. This patient's compulsive gambling and consequently heightened anxiety led to frequent adrenal crisis episodes, which can be fatal if not promptly identified and managed. Initially, the patient was too embarrassed to disclose her symptoms, delaying the recognition of the connection to cabergoline. After her mother had raised concerns about the potential side effects of the medication, the patient discontinued cabergoline, resulting in the rapid resolution of her compulsive behaviors, anxiety, and adrenal crisis episodes. Greater awareness and proactive monitoring for ICDs are crucial, particularly as current hyperprolactinemia and acromegaly management guidelines do not address these risks [16, 17].

Management of ICDs in patients on dopamine agonists involves either discontinuation or dose reductions of the agent, which often leads to rapid resolution of symptoms, as observed in our patient. However, this can sometimes lead to dopamine agonist withdrawal syndrome, characterized by severe physical and psychological symptoms that include anxiety, panic attacks, dysphoria, depression, agitation, irritability, suicidal ideation, fatigue, orthostatic hypotension, nausea, vomiting, diaphoresis, generalized pain, and drug cravings [18]. Alternative medical therapies, such as somatostatin analogs or growth hormone receptor antagonists, may be considered in the management of acromegaly while avoiding dopamine agonist–related side effects [17]. Psychiatric evaluation by a specialist with expertise in ICDs should also be considered, if needed. It has been reported that selective serotonin reuptake inhibitors might be a useful option for treatment of ICDs, as well as cognitive behavioral therapy and psychotherapy [19]. Nevertheless, more data is needed on the efficacy of these approaches to allow for the development of evidence-based guidelines focusing on the management of ICDs induced by dopamine agonist therapy.

## Learning Points

- Dopamine agonists such as cabergoline can cause ICDs, even at the lower doses used in the treatment for prolactinomas and acromegaly. These effects are often under-reported and, therefore, under-recognized.
- ICDs can develop insidiously and may be incorrectly identified as primary psychiatric or psychosocial issues. In individuals with comorbidities like adrenal insufficiency, such misattribution may exacerbate and increase the risk of adrenal crisis episodes.
- Timely recognition and dose reductions or discontinuation of the dopamine agonist often results in rapid symptom resolution. Clinicians should routinely screen for behavioral changes in patients on cabergoline therapy, regardless of the doses used.

## Contributors

All authors made individual contributions to authorship. K.C.J.Y. was involved in diagnosis and management of this patient and manuscript submission. M.M.-G., S.A., and T.N.T. contributed to clinical data collection and literature review. All authors reviewed and approved the final draft.

## Data Availability

Data sharing is not applicable to this article as no datasets were generated or analyzed during the current study.

## Abbreviations

ICD: impulse control disorder
IGF-1: insulin-like growth factor I
MRI: magnetic resonance imaging
